# Prevalence of chronic hepatitis B in Oro Province, Papua New Guinea

**DOI:** 10.5365/wpsar.2020.11.3.001

**Published:** 2020-12-16

**Authors:** Alice Unah Lee, Luke Mair, Bob Kevin, Lily Gandi, Olive Tarumuri, Caroline Lee, Sue Huntley, David Carl Hilmers

**Affiliations:** a University of Sydney, Concord Repatriation General Hospital, Department of Gastroenterology and Liver Services, Hospital Road Concord, Sydney, Australia.; b Department of Infection and Tropical Medicine, Sheffield Teaching Hospitals, Sheffield, United Kingdom.; c Popondetta General Hospital, Popondetta, Papua New Guinea.; d Siroga Clinic, New Britain Palm Oil Company, Ltd, Popondetta, Papua New Guinea.; e Hepatitis B Free, Sydney, Australia.; f Departments of Internal Medicine and Pediatrics and Center for Space Medicine, Baylor College of Medicine, Houston (TX), USA.

## Abstract

Chronic hepatitis B (CHB) affects over 250 million people worldwide. In Papua New Guinea, the prevalence of CHB has been estimated to be over 8%, and it is a leading cause of death. To address this problem, an alliance was formed between the government of Oro Province, a large private employer and an Australian nongovernmental organization, which established a CHB test and treatment programme. Between 2014 and 2019, rapid hepatitis B surface antigen testing was performed on 4068 individuals in Oro Province. The crude prevalence rate was 12.98% and was significantly higher in males (15.26%) than females (10.94%) (*P* < 0.001). The rate was 4.72% among children aged 10 years and under, 12.81% among women of childbearing age (19–35 years) and 18.48% among health-care workers. These results indicate that the rates of vaccination at birth and later among women of childbearing age and health-care workers must be improved to prevent transmission of CHB.

Hepatitis B is a leading cause of morbidity and mortality worldwide; it is responsible for about 900 000 deaths annually, and nearly 300 million people suffer from chronic hepatitis B (CHB). (*1*) The prevalence of CHB in Papua New Guinea (PNG) has been estimated to be 14.6%; (*2*) however, numerous barriers hinder accurate country-wide accounting, as over 80% of the 8 million inhabitants of PNG live in geographically remote areas with limited access to health services, and strong beliefs in traditional healing foster a distrust of western medicine. CHB is a major cause of morbidity and mortality in PNG and the leading cause of cirrhosis and hepatocellular carcinoma. (*3*) Complications of liver cirrhosis, including ascites and variceal bleeding, are reported by local physicians as among the most common reasons for hospital admission. As a result, population-wide screening is critical, with follow-up vaccination of those who are hepatitis B surface antigen (HBsAg) negative and treatment for those who test positive.

Volunteers from Hepatitis B Free (HBF), an Australian non-profit organization, were invited by community leaders and provincial health officials in Oro Province in PNG to address the gap in vaccination against hepatitis B in remote villages. In 2013, HBF donated rapid test kits for HBsAg and began testing and vaccinating individuals in remote communities. Volunteers from HBF travel regularly to PNG and have established a formal partnership with the Oro provincial government, the provincial health department, Popondetta General Hospital and a private company, New Britain Palm Oil Ltd The company is a large employer in Oro Province and provides health care to employees and families as well as to local non-affiliated patients through a network of health clinics and aid posts. In 2019, tenofovir disoproxil fumarate (TDF), a drug with proven efficacy against CHB, was approved for use by the national Government, and the first patients have been started on TDF according to WHO treatment guidelines, (*4*) while population screening continues in government hospitals, outreach health fairs and company-operated clinics.

This report describes surveillance of the large cohort of patients who have been tested since 2014.

## Methods

Ethical approval for the hepatitis B testing and treatment programme was obtained from the Medical Research Advisory Committee of PNG (MRAC No. 18–13). All people attending routine visits to clinics, at community health fairs and during clinical evaluation of symptoms such as abdominal pain were considered eligible and were screened for HBsAg during the period May 2014–October 2019. They were informed of the reason for screening by a health-care worker fluent in their native language and were given the opportunity to ask questions and refuse testing. Those who tested positive were counselled about the risk of transmission, precautions to take and future treatment options and were referred to hepatitis clinics for evaluation. A WHO pre-approved rapid HBsAg test, SD Bioline (Abbott Corp., USA), was used. The reported sensitivity and specificity of this test are 100% and 98.7%, respectively. (*5*) Univariate analyses for gender and HBsAg positivity were performed with χ^2^ tests, and *P* = 0.05 was considered significant. Subgroup analyses were performed after stratification by age, gender and population.

## Results

**Figure 1:**
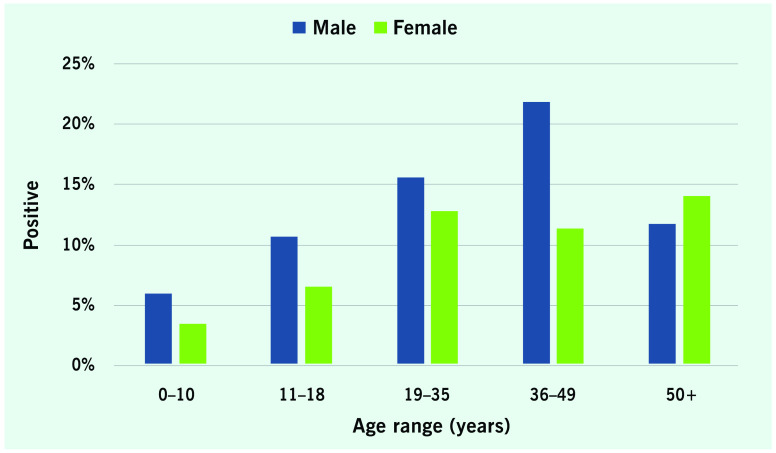
Proportions of persons positive for hepatitis B surface antigen, by age and gender

Between 2014 and the end of 2019, 4068 tests were performed. The refusal rate could not be calculated precisely but is estimated to be < 10%. The overall prevalence of HBsAg positivity was 12.98% (**Table 1**). Males were more likely than females to have positive results (*X*^2^ 1, *n* = 4068) = 16.75, *P* < 0.001. The 36–49 years age group had the highest prevalence (16.90%), and that of men in this age range was 21.90%. The rate was 3.29% among children under 5 years, 4.72% for those under 10 years and 7.06% for those aged 5–15 years. The prevalence among women of prime childbearing age (19–35 years) was 12.81%, while that among women aged 36–49 years was 11.41%, and the rate among girls aged 11–18 years was 6.55%. Of the 92 health-care workers tested, 17 had positive results (18.48%) (**Fig. 1**).

[**insert** Figure 1]

**Table 1:** Numbers and percentages of persons positive for hepatitis B surface antibody, by age and gender

Age range (years)	Positivity				
	Males		Females		Total (%)
	Total (no.)	Positive (no. (%))	Total (no.)	Positive (no. (%))	
0–10	233	14 (6.01)	233	8 (3.43)	4.72
11–18	131	14 (10.69)	229	15 (6.55)	8.06
19–35	934	146 (15.63)	1132	145 (12.81)	14.09
36–49	452	99 (21.90)	412	47 (11.41)	16.90
^3^50	170	20 (11.76)	142	20 (14.08)	12.82
Total	1920	293 (15.26)	2148	235 (10.94)	12.98

Of 4068 individuals tested, only 1134 had either documentation of vaccination or could recall that they had been vaccinated. In this group of 1134, 256 (11.29%) were HBsAg positive. Among children under 5 years, 118 were known to have been vaccinated, but 5 (4.31%) were HBsAg positive.

## Discussion

Our results show high rates of CHB in Oro Province, despite recent efforts to increase vaccination and public awareness. The overall prevalence of 12.98% is lower than previous estimates (14.6%). (*6*) As seen elsewhere, (*7*) we found higher rates among men than women, with the highest prevalence among men aged 36–49 years (21.90%).

The three-dose HBV vaccine was included in the national immunization schedule in 1989, and vaccination at birth was added in 1992. (*8*) A nationwide, four-stage cross-sectional cluster survey among 2109 children aged 4–6 years during 2012–2013 showed an HBV seroprevalence of 2.3%, which is higher than the WHO Western Pacific regional goal of < 1% for children under 5 years. (*9*) In our study, the prevalence was 3.29% among children under 5 years and 4.72% in children under 10 years, which are also higher than the WHO goal. The coverage of vaccination against HBV at birth in PNG is only 31% because of factors such as lack of vaccine and of adequate refrigeration. (*9*) As many women give birth at home without a skilled attendant, timely delivery of a birth dose is difficult. Another study concluded that lack of knowledge about the birth dose among health workers contributed to delay in giving the vaccine. (*10*)

The high prevalence of HBV among adolescent girls, women of childbearing age and health-care workers indicates that testing and vaccination should be improved. Health-care workers are a priority, as they are at risk for both infection and transmission to patients. Universal testing of pregnant women is essential, and antenatal treatment for CHB with new protocols should be considered to decrease the risk of vertical transmission.

Other high-risk groups, including people with HIV and tuberculosis, are not routinely screened for HBV. The prevalence of HIV infection is 0.9% in the general adult population and higher in high-risk groups. (*3*) It is presumed that co-infection with HBV is significant, but testing is often not performed because of lack of availability of rapid testing kits.

It is unclear why CHB appeared to be so prevalent (11.29%) among people who reported previous vaccination. Some may have received fewer than the recommended three doses, mis-reported the type of vaccine administered or were vaccinated when they were already infected but did not know their status. Some may have become infected despite having been vaccinated.

The strengths of this study include the large cohort, the inclusion of individuals in remote areas and use of a sensitive rapid test kit. The limitations include opportunistic testing, possible recall bias of vaccination status and lack of data on co-infection with HIV, hepatitis C virus or tuberculosis. The refusal rate was difficult to calculate; if it was high, the representativeness of the sample would have been biased. As testing was limited to a single province, the results cannot be generalized to the national population.

An antiviral medication, TDF, has been approved for use in PNG. The first patients were started on treatment in Oro Province in November 2019 through the consortium described above. Travel restrictions due to COVID-19 and lack of reagents have slowed the required pre-treatment evaluations, and, thus far, only 20 patients are currently on medication. Consideration should be given to extending treatment to HBV-positive pregnant women. Public education, community HBsAg screening and birth-dose vaccination, which have been prioritized in Oro Province, must be implemented nationwide to achieve the WHO goal of elimination of hepatitis B by 2030.

## Conclusions

We found high rates of CHB in the general population, especially among children under 5 years, women of child-bearing age and health-care workers. The data should assist local and national stakeholders in designing policies and guidelines for therapy and for the prevention of CHB, including vaccination of newborns and at-risk groups, and consideration of prophylactic treatment of HBsAg-positive pregnant women to prevent vertical transmission of hepatitis B. The results should be used to inform policy-makers, mobilize resources and encourage funding from internal and external organizations to reduce the burden of CHB in PNG.
